# Phenotypic variation of cassava root traits and their responses to drought

**DOI:** 10.1002/aps3.1238

**Published:** 2019-04-10

**Authors:** Jitrana Kengkanna, Phissinee Jakaew, Suwaluk Amawan, Natalie Busener, Alexander Bucksch, Patompong Saengwilai

**Affiliations:** ^1^ Department of Biology Faculty of Science Mahidol University Rama VI Road Bangkok 10400 Thailand; ^2^ Rayong Field Crops Research Center Huai Pong Muang Rayong Rayong 21150 Thailand; ^3^ Department of Genetics University of Georgia 120 West Green Street Athens Georgia 30602 USA; ^4^ Department of Plant Biology University of Georgia 120 Carlton Street Athens Georgia 30602 USA; ^5^ Warnell School of Forestry and Natural Resources University of Georgia 180 East Green Street Athens Georgia 30602 USA; ^6^ Institute of Bioinformatics University of Georgia 120 West Green Street Athens Georgia 30602 USA

**Keywords:** cassava, digital imaging of root traits (DIRT), drought, root, shovelomics

## Abstract

**Premise of the Study:**

The key to increased cassava production is balancing the trade‐off between marketable roots and traits that drive nutrient and water uptake. However, only a small number of protocols have been developed for cassava roots. Here, we introduce a set of new variables and methods to phenotype cassava roots and enhance breeding pipelines.

**Methods:**

Different cassava genotypes were planted in pot and field conditions under well‐watered and drought treatments. We developed cassava shovelomics and used digital imaging of root traits (DIRT) to evaluate geometrical root traits in addition to common traits (e.g., length, number).

**Results:**

Cassava shovelomics and DIRT were successfully implemented to extract root phenotypes, and a large phenotypic variation for root traits was observed. Significant correlations were found among root traits measured manually and by DIRT. Drought significantly decreased shoot dry weight, total root number, and root length by 84%, 30%, and 25%, respectively. High adventitious root number was associated with increased shoot dry weight (*r* = 0.44) under drought.

**Discussion:**

Our methods allow for high‐throughput cassava root phenotyping, which makes a breeding program targeting root traits feasible. We suggest that root number is a breeding target for improved cassava production under drought.

Achieving higher edible yields over the next decade is crucial to address the challenge of sustaining a growing human population that faces the consequences of ongoing climate change (Godfray et al., 2010). Current projections suggest that edible yield from all annual crops has to double by 2050 to sustain the growing human population (Jaggard et al., 2010; Tilman et al., 2011). In particular, the rapid expansion of edaphic and drought stresses (Evans, 1998; Santos‐Medellín et al., 2017) poses an acute challenge for plant scientists and breeders to develop crops with higher yields (Rötter et al., 2015). Severe drought stress is identified as a yield‐limiting factor for 47% of the global population, primarily affecting Asia and Africa (Organisation for Economic Co‐operation and Development, 2008). Genetic improvement of crop root architecture has been identified as a target (de Dorlodot et al., 2007) to develop more productive and stress‐tolerant crops (Hall and Richards, 2013).

Cassava (*Manihot esculenta* Crantz) is a tropical root crop that can be produced efficiently on a small scale, without the need for mechanization or purchased fertilizer inputs (Fasinmirin and Reichert, 2011). As such, genetic improvement of cassava is identified as a target for ensuring food security (Sheffield et al., 2006) and reducing poverty (Leunufna and Evans, 2014). The economic impact of cassava influences developing countries like Thailand, which is the world's largest exporter of dried cassava root (Hillocks and Thresh, 2001). Despite cassava's enormous capability to tolerate drought conditions, the increasing severity of drought events challenges further improvements of cassava root yields (Bakayoko et al., 2009). Several studies have shown an increased susceptibility of cassava to drought during the first three months after planting (El‐Sharkawy, 2007). Drought conditions were reported to consistently reduce root yields by about 32% compared with well‐watered crops (Connor et al., 1981), and prolonged water stress lasting five months further increases yield loss to over 60% (Oliveira et al., 1982). Consequently, there is a pressing demand for phenotyping solutions that help breeders develop cassava genotypes that provide sufficient yield under drought conditions.

Improving root systems has been shown to be a successful strategy to enhance water and nutrient uptake in important agronomic crops (Lynch, 1995, 2013; Borch et al., 1999; Postma and Lynch, 2012; York et al., 2013; Paez‐Garcia et al., 2015). Root traits contributing to deep root systems such as low crown root number (Saengwilai et al., 2014b), high root cortical aerenchyma (Saengwilai et al., 2014a), and reduced lateral root branching (Zhan et al., 2015) have been demonstrated to enhance water and nitrate uptake, whereas traits promoting shallow root systems such as increased adventitious root number and lateral root development (Burridge et al., 2016) have been shown to improve uptake of immobile nutrients such as phosphorus in dicots (Burridge et al., 2016). Despite the benefits of improving root traits associated with nutrient and water uptake, breeding programs often consider the development of root traits as infeasible for many crops because of a twofold knowledge gap: (1) What are the important traits for a given crop? and (2) How can belowground traits be phenotyped?

Less is known about the development of cassava roots compared to other crops, because cassava is typically propagated from woody stem cuttings in agricultural production systems. In the case of stem cuttings, adventitious roots emerge at the basal region of the stem cut and develop the shoot system through axillary bud growth (Medina et al., 2007). Adventitious root formation can then be divided into two types: (1) basal roots formed at the cut stem ends that developed from the cambium and (2) nodal roots initiated in deeper tissues surrounded by xylem and pith at the axillary bud (Chaweewan and Taylor, 2015). Later in development, root bulking occurs when photosynthates produced in the developing canopy are diverted and translocated from the shoot to the adventitious roots. As a result, adventitious roots transform into specialized storage root organs to store starch (Alves, 2002). A noteworthy strong morphological variation can be observed in storage roots, which poses challenges and opportunities in the phenotypic description of the cassava root system (Chaweewan and Taylor, 2015). However, only a small number of protocols that record a limited number of cassava root traits have been developed so far (Adjebeng‐Danquah et al., 2016a, b). In particular, geometric root traits such as root area and width have been neglected in current protocols (Okogbenin et al., 2013). Hence, a better understanding of morphological variation in cassava roots will expand opportunities to improve breeding programs and agronomic management.

In recent years, a number of automated root phenotyping approaches for cereals and annual crops such as maize, barley, wheat, rice, and legume have been developed (Lobet et al., 2013). A broad range of manual and 2D/3D‐imaging phenotyping platforms allow for the measurements of root traits from plant seedlings to mature plants in the field. A few examples of these methods include rhizoponics (Mathieu et al., 2015), X‐ray computed tomography and magnetic resonance imaging (Metzner et al., 2015), GLO‐Roots (Rellán‐Álvarez et al., 2015), rhizoslides (Le Marié et al., 2014), minirhizotrons (Iversen et al., 2012), and shovelomics (Trachsel et al., 2011). Root architectural traits such as root angle and crown root number can be quickly evaluated with shovelomics. Therefore, shovelomics has been widely adopted for physiological and genetic studies of a wide variety of crops (Atkinson et al., 2019). Coupled with phenotyping platforms, several software packages have been developed to process thousands of root architectural and anatomical images. Digital imaging of root traits (DIRT) is an online platform for plant phenotyping that researchers can freely use to analyze digital pictures of plant roots (Das et al., 2015). DIRT has high throughput and can compute more than 70 phenotypic traits of fibrous monocot and dicot roots (Bucksch et al., 2014). Moreover, it is an open‐source phenomics platform that allows collaboration and sharing of root phenomics data with other researchers worldwide. Currently, DIRT has been used to phenotype root traits of many plant species including maize (Bray and Topp, 2018), cowpea (Burridge et al., 2017), and common bean (Burridge et al., 2016), but it has never been applied to tuber and root crops.

Our phenotyping approach uses both manual evaluation and DIRT image analysis to observe the phenotypic variation of cassava root traits of three‐, 10‐, and 12‐month‐old cassava from the Thai germplasm. A set of geometric root traits that relate to plant performance under drought was quantified by DIRT. We identified specific cassava root traits that correlate with increased water use efficiency. Hence, our results pave the way for new cassava varieties bred for high marketable yields under drought.

## METHODS

### Plant materials and growth conditions

Experiments were carried out in pot growth systems and under field conditions. The pot system experiment included five cassava genotypes: Rayong 5 (R5), Rayong 9 (R9), Rayong 11 (R11), Huay Bong 60 (HB60), and Kasetsart 50 (KU50). These genotypes were chosen to encompass the differences in root lengths (long roots: R9, HB60, and R11; short roots: KU50 and R5). All experiments were planted using 20‐cm stem cuttings obtained from the Rayong Field Crops Research Center (Rayong, Thailand). In the field, seven common Thai genotypes (R5, R9, R11, Rayong 86‐13 [R86‐13], HB60, Huay Bong 80 [HB80], and KU50) were investigated for phenotypic variation of root traits. R5, R9, and R11 were further selected for a drought field trial because they produced a high number and weight of storage roots. Moreover, they are recommended by the Thailand Department of Agriculture as drought‐tolerant candidates (http://at.doa.go.th/cassava/variety_cas.phpURLHASH;).

### Growth conditions

The pot experiment was conducted at Mahidol University (Salaya campus) in Nakhon Pathom, Thailand (13°47′40.2″N, 100°19′26.7″E), from November 2016 to January 2017. The stem cuttings were placed vertically in the soil such that two‐thirds of the stem was below the soil line. We used 24‐cm‐tall white plastic pots with a diameter of 25 cm at the top and 17 cm at the bottom. Each pot contained 5 kg of organic growth media containing rain tree leaf soil and bamboo soil (1 : 1 volume ratio). Water‐holding capacity was measured and processed as described by Noggle and Wynd (1941), and it was calculated as the percentage from the ratio of mass of the water in saturated soil to the mass of the saturated soil. The soil in each pot was saturated with water and drained for a day to evaluate percentage of soil water content that was determined by gravimetric measurement (Evett, 2008). We found that the water‐holding capacity in the pot system was 52.48% and the soil water content was 83%. We planted five biological replicates under well‐watered and drought conditions. The plants were placed outside for 30 days and were watered every other day with 1 L of water. Twenty‐four days after planting (DAP), 20 g of fertilizer containing 16% each of nitrogen, phosphorus, and potassium (16‐16‐16) per pot was applied. According to Adu et al. (2018), cassava has the highest relative growth rate during the first 30 DAP and the growth rate is subsequently decreased until nearly constant after 30 to 60 DAP; therefore, drought treatment was applied at 30 DAP to allow time for plants to acclimate to the system and yield information relevant to developmental processes. All plants were transferred into a growth shelter made of transparent plastic sheets to protect the plants from rainwater. To simulate drought conditions, half of the plants received no water from 30 to 90 DAP, after which they were harvested and phenotyped. The total amount of water that the plants received in the well‐watered and drought treatments were approximately 45 L and 15 L, respectively. Soil water content decreased to 46.95% and 28.36% at seven and 14 days after drought treatment, respectively. At this time, the plants showed significant reduction in height and number of leaves under drought in all genotypes.

Two field experiments were conducted at the Rayong Field Crops Research Center, Mueng, Rayong, Thailand (12°44′01″N, 101°08′02″E). For the first trial, the seven genotypes were grown in loamy sand soil from April 2015 to February 2016 under rainfed conditions. The field capacity was approximately 14%. The total amount of rainfall during this season was 1517.9 mm, with drought periods from April to May. We planted a randomized complete block design with four replications. In the second trial, three genotypes (R5, R9, and R11) were planted under well‐watered and drought treatments between April 2016 to April 2017. The total amount of rainfall during this experiment was 1714.3 mm. Each plot had 700 plants, and seven replicates were harvested in this trial. The distance between rows and between plants was 1 m. The plants were watered once per week in the first two months. We followed the fertilization regime recommended by the Thailand Department of Agriculture. This regime applies 87.5 kg·ha^−1^ of urea, 54.81 kg·ha^−1^ of diammonium phosphate (18‐46‐0), and 271.56 kg·ha^−1^ of potassium chloride (0‐0‐60). After two months of growth, well‐watered plants received irrigated water every other day, while the drought treatment plants were rainfed and did not receive additional water. Shoot and root traits were quantified at 10 months (first trial) and 12 months (second trial) after planting.

### Data collection

**Figure 1:**
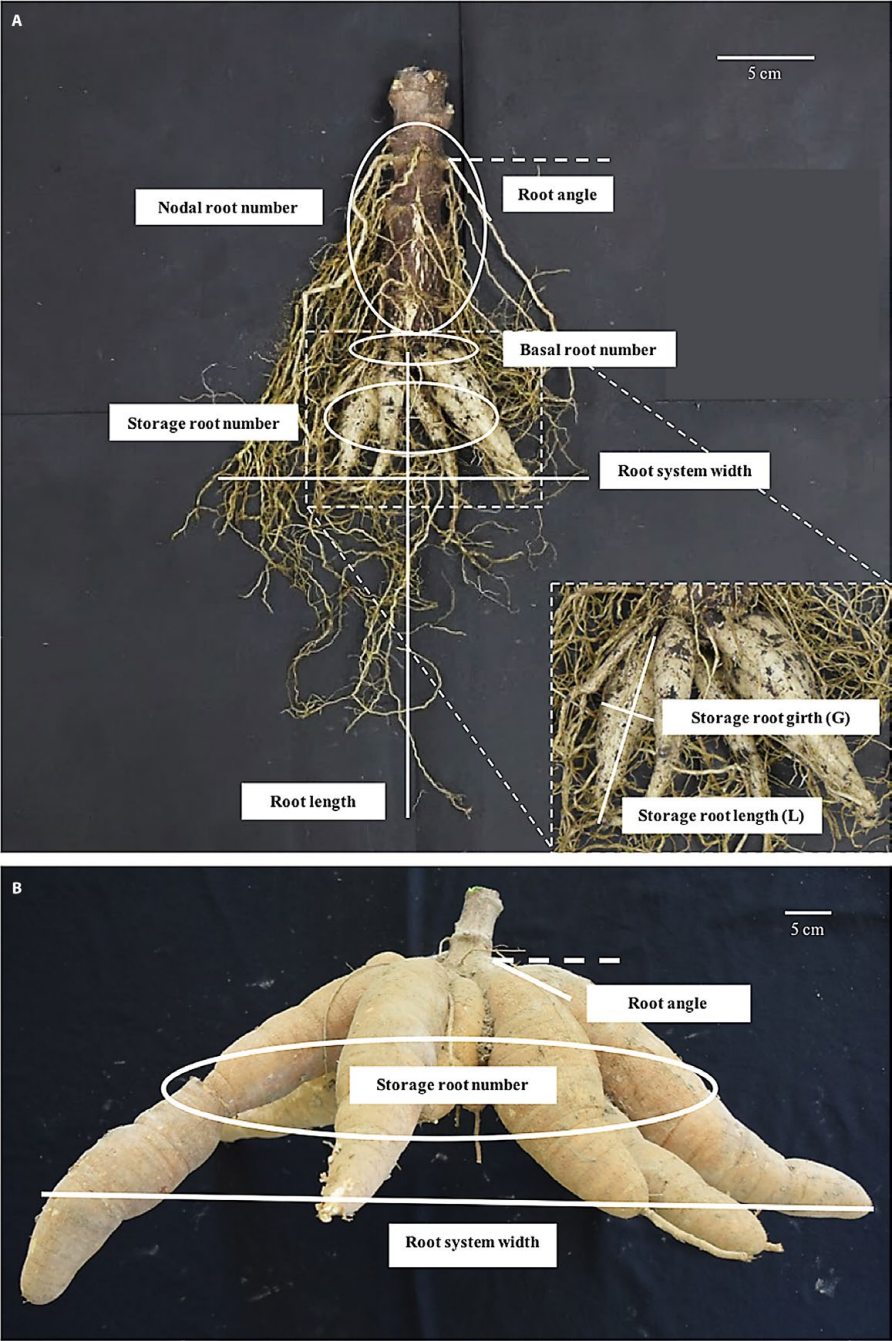
Cassava root traits assessed manually using the excavated root crowns of plants at three months (A) and 10 months (B) after planting.

**Figure 2:**
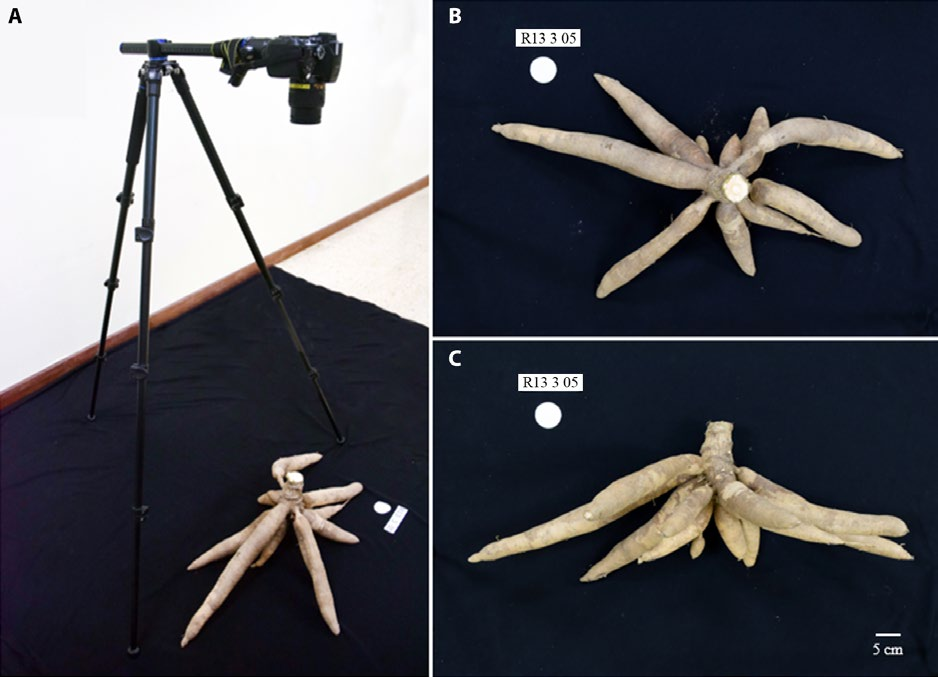
The setup for capturing cassava root images used in this study. (A) Imaging setup consists of a large black cloth, a camera (Nikon D5300; Nikon, Tokyo, Japan) with a tripod, a 2‐in‐diameter circle scale, and a label. Images were captured from top view (B) and side view (C).

Manual evaluation was carried out using a modified shovelomics approach (Trachsel et al., 2011). Modifications to the original shovelomics protocol include the use of a handheld brush to clean sand and soil particles from the root system instead of ringing with water. Overall, we measured 19 cassava traits including shoot and root traits for the pot experiment and 12 traits for the field experiments using a newly developed phenotyping protocol. The trait measurements are specialized for perennial plants with large storage root morphologies and shown in Table 1. The number of basal roots and nodal roots were quantified by counting manually. The basal root length was measured using a standard ruler. The shape of the storage root system was characterized by measuring the widest part of a representative root as storage root girth and the longest individual root length as storage root length (Fig. 1A). In addition, we counted the number of storage roots, measured the largest extension of the whole root system as the root system width, and evaluated root angle by using a shovelomics board (Trachsel et al., 2011) for both pot and field experiments (Fig. 1B). The imaging station was set up to capture pictures of the root system for DIRT (Fig. 2). Two photos of top and side view were used for root trait analysis. Our imaging setup consists of large black cloth, a digital camera (Nikon D5300; Nikon, Tokyo, Japan) with tripod, a white circle two inches in diameter, and tags to record genotype and treatment in the captured image. Overall, we took two images per root system: top view (Fig. 2B) and side view (Fig. 2C). The images were uploaded to the DIRT website for automatic trait extraction (http://dirt.cyverse.org/). We used the following DIRT traits to validate and interpret cassava root morphology: median width (WIDTH_MED) and maximum width (WIDTH_MAX) of the root system, width derived from a medial axis transformation (SKL_WIDTH), rooting angle, root system area (AREA), stem diameter (DIA_STEM), rooting depth (SKL_DEPTH), percentage of total accumulated width at depth levels ranging from 10–90% (D10‐90), and the rate at which root system width accumulates at depth levels ranging from 10–90% (DS10‐90) (Table 1).

**Table 1:** Overview of variables measured by cassava shovelomics and DIRT of cassava shoot and root traits. Trait names are listed with description and physiological interpretation

Traits	Organ	System	Description	Physiological interpretation	Reference
**Shovelomics measurements**					
Plant height (cm)	Shoot	Pot/field	Measure from the base of a stem to the highest point of a shoot	Light interception level and growth	Mathan et al., 2016
No. of branches	Shoot	Pot	Count number of branches	Light interception level and growth	Mathan et al., 2016
No. of leaves	Shoot	Pot	Count number of leaves	Photosynthetic activity and growth	Mathan et al., 2016
Stem diameter (cm)	Shoot	Pot/field	Measure a stem diameter at 2 cm above the root	Xylem water potential and growth	Genard, 2001
Shoot dry weight (g)	Shoot	Pot	Weigh dry shoots after being dried in an oven at 70°C for 48 h with a balance	Biomass	
Shoot weight (kg)	Shoot	Field	Weigh fresh shoots with a balance	Biomass	
Average root angle (degree)	Root	Pot/field	Measure left and right angle with a shovelomics scoreboard	Root nutrient foraging	Trachsel et al., 2013
Root system width (cm)	Root	Pot/field	Measure the width of a root system with a ruler	Root nutrient foraging	Subere et al., 2009
Root length (cm)	Root	Pot	Measure the length of basal roots with a ruler	Root nutrient foraging	Lynch, 2013
No. of basal roots	Root	Pot	Count number of basal roots	Soil exploration volume	
No. of nodal root	Root	Pot	Count number of nodal roots	Soil exploration volume	
No. of adventitious roots	Root	Pot/field	Total number of basal and nodal roots	Soil exploration volume	Alves, 2002
No. of storage roots	Root	Pot/field	Count number of storage roots	Soil exploration volume and yield	Alves, 2002
Total no. of roots	Root	Pot	Total number of basal, nodal, and storage roots	Soil exploration volume	Alves, 2002
Storage root girth (G) (cm)	Root	Pot/field	Measure the width of a storage root using a vernier caliper	Photosynthate accumulation pattern	Adjebeng‐Danquah et al., 2016a
Storage root length (L) (cm)	Root	Pot/field	Measure the length of a storage root with a ruler	Photosynthate accumulation pattern	Adjebeng‐Danquah et al., 2016a
Ratio L : G	Root	Pot	Ratio between L and G	Photosynthate accumulation pattern	Adjebeng‐Danquah et al., 2016a
Storage root circumference (cm)	Root	Pot/field	Measure circumference of storage root with a tape measure	Photosynthate accumulation pattern	
Storage root weight (g)	Root	Field	Weigh storage roots with a balance	Yield	
Root dry weight (g)	Root	Pot	Weigh dry roots after being dried in an oven at 70°C for 48 h with a balance	Biomass	
Plant dry weight (g)	Shoot/root	Pot	Weigh dry shoot, stem, and root with a balance	Biomass	
Plant weight (kg)	Shoot/root	Field	Weigh fresh shoot, stem, and root with a balance	Biomass	
**DIRT measurements**					
DIA_STEM	Shoot	Pot/field	Stem diameter derived from the medial axis	Xylem water potential	Genard, 2001
WIDTH_MED	Root	Pot/field	Median width of root system measured horizontally	Root nutrient foraging	Subere et al., 2009
WIDTH_MAX	Root	Pot/field	Maximum width of root system measured horizontally	Root nutrient foraging	Subere et al., 2009
SKL_WIDTH	Root	Pot/field	Skeleton width calculated from the medial axis	Root nutrient foraging	Subere et al., 2009
Root angle	Root	Field	Average root angle of left and right angles of root system	Root nutrient foraging	Trachsel et al., 2013
AREA	Root	Pot/field	Projected root area	Soil exploration volume and yield	
SKL_DEPTH	Root	Pot/field	Rooting depth skeleton calculated from the root‐tip path (RTP) skeleton	Root nutrient foraging	Lynch, 2013
D 10*‐*90	Root	Field	Accumulated width over the depth at x***%*** of the central path length. The change in width accumulation denotes a change of the root top angle.	Root nutrient foraging	Bucksch et al., 2014
DS 10*‐*90	Root	Field	Slope of the graph of central path length vs. accumulated width at x% of the accumulated width	Root nutrient foraging	Bucksch et al., 2014

### Statistical analysis

R version 3.2.1 (R Core Team, 2014) was used to perform ANOVA analyses with the *agricolae* package (Mendiburu, 2010). We assumed genotypes and treatments as independent variables to compare trait variation among five cassava genotypes in well‐watered and drought treatments. Measured root and shoot traits were assumed to be dependent variables in our analysis. One‐way ANOVA was used to compare the traits of all seven genotypes grown in the field. The protected least significant difference (LSD) post hoc test (α = 0.05) was used as a multiple comparison test. Pearson correlation, by the *PerformanceAnalytics* package, was used to determine relationships between cassava traits of the seven genotypes grown in the field under rainfed conditions and the five genotypes grown in pots under drought conditions. In addition, to perform the validation of DIRT traits with manual measurements, we used Python 2.7.13 (Oliphant, 2007) with the *scikit‐learn* 0.19.1 (Pedregosa et al., 2011) package to perform the RANSAC regression as well as the *SciPy* package (Jones et al., 2001) to compute standard linear regressions. The regression plots were generated with the matplotlib library, and the normalized mean value comparisons were plotted with plotly.

## RESULTS

### Root traits show strong phenotypic variation among Thai cassava genotypes

Manual root phenotyping revealed considerable variation for root traits among different cassava genotypes. At three months in the pot system, phenotypic variation ranged from 1.6‐fold in stem diameter to 40‐fold in nodal root number under the well‐watered treatment (Table 2). At 10 months in the field, the range of variation was substantial: 2.72‐fold in root system width, 3.57‐fold in storage root number, fourfold in root angle, and 7.68‐fold in storage root weight (Table 3). Among genotypes, R5 had the highest average storage root weight and plant weight as well as the highest root angle. Correlation analysis revealed significant positive correlations between root system width and other traits, including plant weight (*r* = 0.58, *P* < 0.01), storage root weight (*r* = 0.61, *P* < 0.001), and storage root number (*r* = 0.35, *P* < 0.05), whereas root angle significantly correlated with plant weight (*r* = 0.33, *P* < 0.05) and storage root weight (*r* = 0.36, *P* < 0.05) (Table 4). In addition, storage root number was significantly correlated with plant weight (*r* = 0.56, *P* < 0.01) and storage root weight (*r* = 0.44, *P* < 0.05).

**Table 2:** Phenotypic variation of traits among five cassava genotypes under two treatments: well‐watered (W) and drought condition (D) using the pot system.^a^

Traits	Treatment	Genotypes					Average	Range
		R5	R9	R11	HB60	KU50		
Height (cm)	W	77.5 ± 0.78a	72.2 ± 1.31a	73.6 ± 0.88a	73.2 ± 0.81a	78.8 ± 0.73a	75.0 ± 0.02*	1.7
	D	55.4 ± 0.85a	24.7 ± 0.38c	24.4 ± 0.56c	40.6 ± 0.58b	39.13 ± 1.11b	36.8 ± 0.06	3.8
No. of branches	W	2 ± 0.00a	2.8 ± 0.11a	3.2 ± 0.08a	1.8 ± 0.08a	1.4 ± 0.05a	2.25 ± 0.02	1.8
	D	2 ± 0.12a	3.6 ± 0.17a	2.8 ± 0.08a	3 ± 0.12a	2.75 ± 0.21a	2.83 ± 0.03	5.0
No. of leaves	W	79.5 ± 0.78a	75 ± 1.30a	76.8 ± 0.89a	75 ± 0.83a	80.2 ± 0.70a	77.2 ± 0.18*	1.6
	D	57.4 ± 0.73a	28.3 ± 0.42c	27.2 ± 0.53c	43.6 ± 0.48b	41.88 ± 1.04b	39.6 ± 0.27	3.1
Average root angle (degree)	W	32 ± 2.06ab	40.8 ± 1.64a	23 ± 0.84b	31.4 ± 1.32ab	25.6 ± 0.56ab	30.5 ± 0.27	5.8
	D	29.6 ± 0.93ab	42.67 ± 1.00a	26.5 ± 2.62ab	18.6 ± 0.81b	30 ± 1.53ab	28.5 ± 0.30	7.3
Root system width (cm)	W	15.88 ± 0.15a	15.16 ± 0.34a	16.72 ± 0.37a	15.1 ± 0.24a	16.08 ± 0.24a	15.8 ± 0.05*	2.0
	D	11.92 ± 0.3ab	11 ± 0.18ab	5.95 ± 0.06b	13.46 ± 0.57a	12.5 ± 0.51ab	11.7± 0.09	3.7
Adventitious root length (cm)	W	23.78 ± 0.29a	26.66 ± 1.20a	23 ± 0.68a	26.26 ± 0.81a	29.94 ± 0.78a	26.0 ± 0.17*	2.9
	D	19.04 ± 0.51a	19.25 ± 1.03a	20.17 ± 1.41a	19.58 ± 0.20a	20.03 ± 0.97a	19.6 ± 0.15	7.5
No. of basal roots	W	16 ± 0.42de	22.4 ± 1.19cd	28.8 ± 0.91bc	26.8 ± 1.1bcd	30.6 ± 0.09bc	25.3 ± 0.19	3.1
	D	27.8 ± 0.64b	10.2 ± 0.93c	9.4 ± 1.31c	38 ± 1.25ab	46.75 ± 0.61a	25.6 ± 0.36	54
No. of nodal roots	W	13.75 ± 0.6bc	20.2 ± 0.91ab	25.4 ± 0.52a	12.2 ± 0.58bc	3.6 ± 0.20d	15.1 ± 0.23*	40.0
	D	8 ± 0.16abc	11 ± 0.42a	8.6 ± 0.31ab	4.8 ± 0.26bc	4.5 ± 0.26c	7.5 ± 0.08	18
No. of adventitious roots	W	29.8 ± 0.8cde	42.6 ± 0.75ab	54.2 ± 0.98a	39 ± 1.69bc	34.2 ± 0.27cd	40.4 ± 0.26	3.9
	D	35.8 ± 0.55ab	21.2 ± 1.16bc	18 ± 1.54c	42.8 ± 1.28a	51.25 ± 0.66a	33.1 ± 0.34	14.8
No. of storage roots	W	14.5 ± 0.22a	6.2 ± 0.28b	1.4 ± 0.09c	7.8 ± 0.30b	7 ± 0.21b	7.00 ± 0.10*	16.0
	D	0.6 ± 0.09a	0.00 ± 0.00a	0.00 ± 0.00a	0.2 ± 0.05a	0.5 ± 0.07a	0.25 ± 0.01	2
Total no. of roots	W	44.3 ± 0.9abc	48.8 ± 0.7abc	55.6 ± 0.90a	46.8 ± 1.6abc	41.2 ± 0.50bc	47.5 ± 0.21*	2.8
	D	36.4 ± 0.53a	21.2 ± 1.16b	18 ± 1.54b	43 ± 1.31a	51.75 ± 0.70a	33.3 ± 0.34	15
Storage root girth (G) (cm)	W	2.18 ± 0.03a	2.68 ± 0.08a	0.94 ± 0.07b	2.47 ± 0.03a	2.57 ± 0.03a	2.22 ± 0.02*	11
	D	0.43 ± 0.01a	—	—	0.4 ± 0.00a	0.365 ± 0.01a	0.4 ± 0.001	1.7
Storage root length (L) (cm)	W	17.05 ± 0.81a	13.53 ± 0.69a	16.20 ± 0.59a	11.60 ± 0.58a	15.61 ± 0.65a	14.6 ± 0.13	4.4
	D	10.1 ± 0.113a	—	—	11.1 ± 0.00a	10.75 ± 0.13a	10.6 ± 0.02	1.2
Ratio L : G	W	7.92 ± 0.41b	4.98 ± 0.19b	32.06 ± 2.64a	4.89 ± 0.29b	5.97 ± 0.21b	10.4 ± 0.30	23.5
	D	24.44 ± 0.83a	—	—	27.75 ± 0.00a	30.04 ± 0.58a	27.3 ± 0.11*	1.8
Storage root circumference (cm)	W	7.05 ± 0.06a	8.3 ± 0.20a	2.8 ± 0.24b	8.14 ± 0.10a	8.34 ± 0.06a	6.92 ± 0.05*	12.5
	D	1.3 ± 0.06a	—	—	1.4 ± 0.00a	1.05 ± 0.08a	1.22 ± 0.01	2.8
Stem diameter (cm)	W	1.943 ± 0.01d	2.788 ± 0.01a	2.3 ± 0.01bc	2.364 ± 0.02b	2.1 ± 0.02bcd	2.32 ± 0.01	1.6
	D	2.306 ± 0.02a	2.068 ± 0.02a	2.056 ± 0.04a	2.32 ± 0.02a	2.25 ± 0.04a	2.2 ± 0.01	1.6
Shoot dry weight (g)	W	38.25 ± 0.5bc	41.96 ± 1.01b	52.78 ± 0.73a	32.27 ± 1.44c	36.82 ± 0.5bc	40.5 ± 0.23*	4.6
	D	8.324 ± 0.22a	6.3 ± 0.17ab	3.872 ± 0.13b	6.488 ± 0.19a	7.725 ± 0.33a	6.49 ± 0.05	5.2
Root dry weight (g)	W	14.35 ± 1.46a	10.12 ± 2.04a	11.58 ± 2.33a	9.251 ± 2.06a	8.499 ± 2.44a	10.61 ± 0.1*	8.8
	D	0.8764 ± 0.16a	0.2325 ± 0.10b	0.2459 ± 0.12b	0.7326 ± 0.10a	0.851 ± 0.16a	0.5767 ± 0.1	107
Plant dry weight (g)	W	80.27 ± 3.55ab	75.73 ± 5.76ab	88.22 ± 5.80a	68.26 ± 7.71b	72.72 ± 5.19ab	76.9 ± 1.2*	2.4
	D	27.14 ± 2.29a	19.85 ± 1.02b	18.44 ± 2.33b	20.97 ± 3.00ab	23.24 ± 3.63ab	21.87 ± 2.8	2.53

a Different letters denote significant differences among genotypes in each treatment (*P* < 0.05), while an asterisk (*) denotes significant difference between treatments of the average value of traits (*P* < 0.05).

**Table 3:** Summary of phenotypic variation of root and shoot traits among seven cassava genotypes grown in the field. The plants were harvested at 10 months after planting.^a^

Genotypes	Root traits				Shoot traits	
	No. of storage roots	Storage root weight (kg)	Root angle (degree)	Root system width (cm)	Plant height (cm)	Plant weight (kg)
R5	17.75 ± 1.11a	7.09 ± 0.56a	27.50 ± 4.79a	54.25 ± 6.74a	241.25 ± 13.28ab	10.24 ± 0.45a
R9	18.50 ± 2.33a	3.29 ± 0.26b	21.25 ± 4.27abc	55.25 ± 5.53a	232.75 ± 4.42b	5.33 ± 0.55b
R11	12.50 ± 2.06bc	2.56 ± 0.57bc	25.00 ± 2.89ab	53.00 ± 2.48a	180.25 ± 5.51c	4.52 ± 0.80b
R86‐13	19.75 ± 2.56a	2.43 ± 0.40bc	25.00 ± 6.45ab	45.00 ± 7.86a	250.75 ± 7.45ab	5.30 ± 0.92b
KU50	10.25 ± 1.18c	3.50 ± 0.87b	15.00 ± 2.89bc	53.50 ± 5.42a	254.00 ± 18.00ab	4.88 ± 0.85b
HB60	9.25 ± 0.63c	1.36 ± 0.19c	11.25 ± 1.25c	44.00 ± 4.14a	272.50 ± 17.97a	2.58 ± 0.23c
HB80	16.50 ± 1.71ab	2.11 ± 0.24bc	12.50 ± 2.50c	45.50 ± 4.99a	273.25 ± 5.45a	3.88 ± 0.33bc
Mean	15.21 ± 0.96	3.19 ± 0.37	19.64 ± 1.76	50.07 ± 2.05	243.53 ± 6.86	5.24 ± 0.48
Range	3.57	7.68	4.00	2.72	1.88	5.30

a Different letters denote significant difference at *P* < 0.05.

**Table 4:** Correlations among cassava traits in 10‐month‐old cassava grown in the field.^a^

Trait	Shoot trait		Root trait		
	Plant height (cm)	Plant weight (kg)	Storage root weight (kg)	No. of storage roots	Root angle (degree)
Plant weight (kg)	0.032				
Storage root weight (kg)	−0.075	0.95***			
No. of storage roots	0.055	0.56**	0.44*		
Root angle (degree)	−0.25	0.33*	0.36*	0.13	
Root system width (cm)	−0.13	0.58**	0.61***	0.35*	0.18

^a^Significant difference at ***P < 0.001, **P < 0.01, and *P < 0.05.

**Figure 3:**
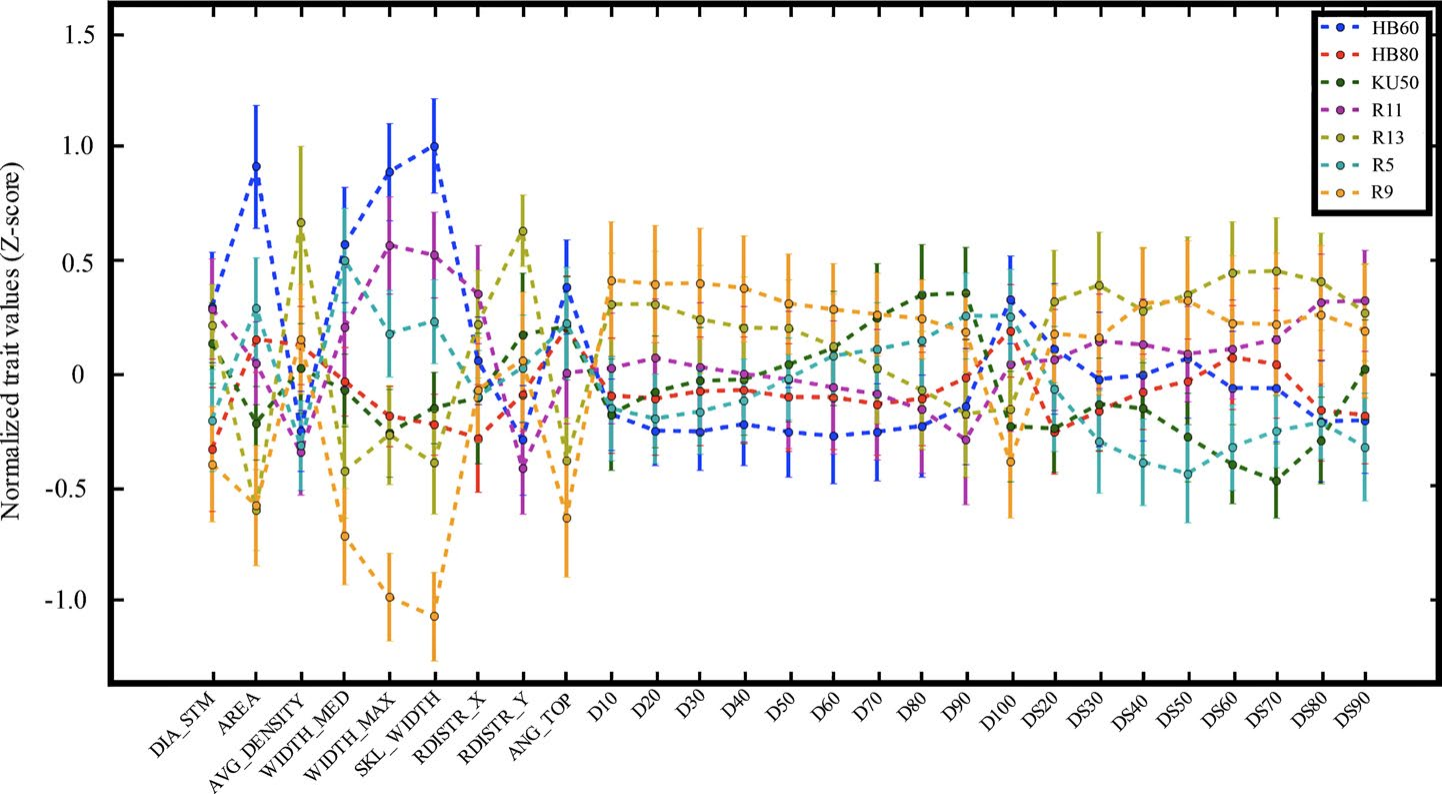
Genotypic discrimination of DIRT traits. Points represent average trait values. Error bars depict the standard error of the mean, and dotted lines guide the reader visually between averages per genotype. Traits were made comparable by calculating the *Z*‐score of each trait.

**Figure 4:**
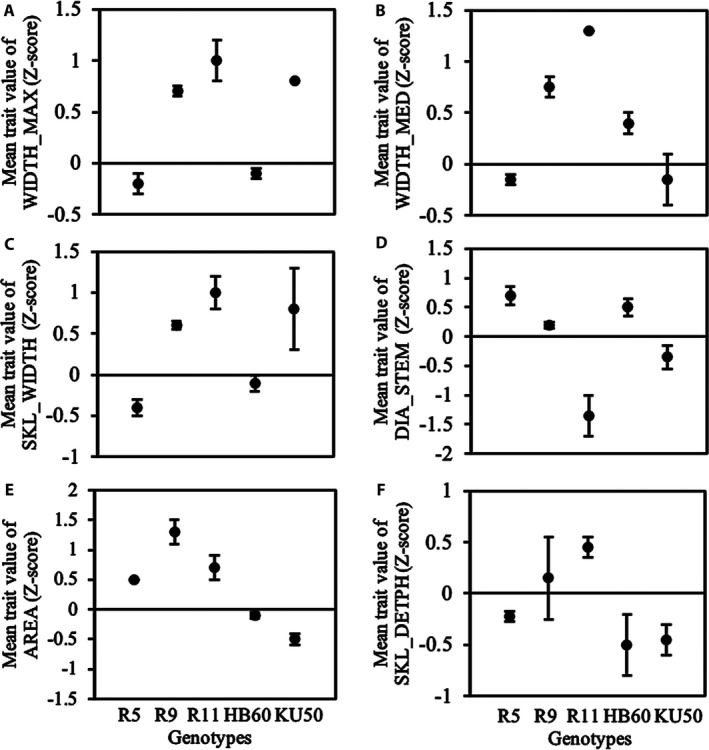
DIRT trait comparison for R5, R9, R11, HB60, and KU50. The plants were harvested at three months after planting in the pot system under well‐watered conditions. (A) WIDTH_MAX, (B) WIDTH_MED, (C) SKL_WIDTH, (D) DIA_STEM, (E) AREA, and (F) SKL_DEPTH. Data were made comparable through normalization of mean trait values (*Z*‐score). Error bars represent the standard error of the mean for each treatment and genotype category that corresponds to a particular trait.

**Figure 5:**
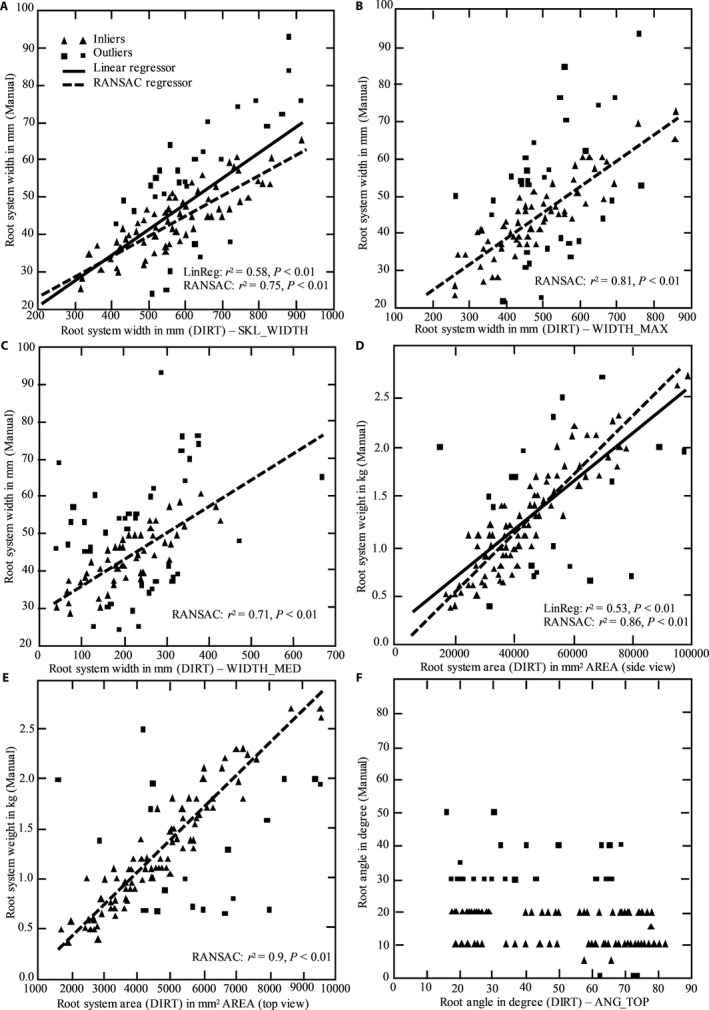
Regression analysis of cassava shovelomics and DIRT traits between the root system width and medial axis‐derived width (SKL_WIDTH) (A), the maximum width (WIDTH_MAX) (B), and the median width (WIDTH_MED) (C). Significant correlations were found between root weight and DIRT traits including AREA of side view (D) and top view (E) images. No correlation was found for root angle (F) when compared between a manual measurement and DIRT analysis.

We observed significant genotypic variation in several root traits using the imaging setup and DIRT for image analysis. Overall, image‐based analysis could distinguish all genotypes by at least four traits. The best discrimination of genotypes could be found in width measurements *(*WIDTH_MED, WIDTH_MAX, SKL_WIDTH*)*, stem diameters *(*DIA_STEM*)*, root area *(*AREA*)*, and root width accumulation at depth levels *(*D, DS values, and SKL_DEPTH*)* as shown in Figures 3 and 4. We performed a regression analysis to compare DIRT with our manual phenotyping protocol. The regression analysis used standard linear regression and an advanced RANSAC regression to account for outliers in both phenotyping protocols. All root width traits yielded very high correlations, with a coefficient of determination greater than 0.7 and *P* values less than 0.01 for the RANSAC regression *(*Fig. 5A–C*)*. The linear regression of the medial axis‐derived width resulted in a coefficient of determination of 0.58 and a *P* value less than 0.01. High correlations for linear regression (*r* ^2^ > 0.5, *P* < 0.01*)* and RANSAC regression (*r* ^2^ > 0.85, *P* < 0.01*)* were achieved for the root weight and the measured root area in DIRT *(*Fig. 5D*)*. Root weight represents the traditionally most important root trait, as it reflects the amount of yield harvested from the root system. Root area could achieve highly significant correlations with root weight *(P* < 0.01*)* from the top and side view of the images and explain up to 60% of the variation present between manual and automatic measurement *(*Fig. 5D, E*)*. The correlation between root angle measured by the manual protocol and by DIRT was not significantly correlated *(*Fig. 5F*) because of differences in the measurement procedure*.

### Effects of drought on shoot and root traits

We found that the progression of drought significantly reduced plant height and leaf number as early as 14 days after starting the drought treatment. At harvest, drought significantly reduced average shoot dry weight by a factor of six. Furthermore, well‐watered cassava resulted in two times greater plant height, leaf number, and nodal root number compared to drought conditions. Drought reduced root system width, total root number, and adventitious root length by 26%, 30%, and 25%, respectively *(*Table 2 *)*. There was no storage root formation under drought except for genotypes R5, HB60, and KU50. Therefore, storage root number, storage root girth, and storage root circumference were reduced by 96%, 82%, and 82%, respectively. Storage root length to girth ratio under drought increased by a factor of 2.6 compared to well‐watered treatments.

Of the different genotypes, R5, HB60, and KU50 increased adventitious root number by 20%, 10%, and 50%, respectively, whereas R9 and R11 decreased adventitious root number by 50% and 67%, respectively, under drought *(*Table 2 *)*. Only KU50 had greater total root number under drought conditions compared to well‐watered treatment. R11 had the lowest shoot dry weight with shoot dry weight reduced 92.7% under drought conditions, followed by R9, HB60, KU50, and R5, for which shoot dry weight was decreased by 85%, 79.9%, 79%, and 78%, respectively *(*Table 2 *)*. In addition, we found that the adventitious root number was positively correlated with shoot dry weight *(r* = 0.44, *P* < 0.05), root dry weight (*r* = 0.70, *P* < 0.001), and plant dry weight (*r* = 0.37, *P* < 0.05).

**Figure 6:**
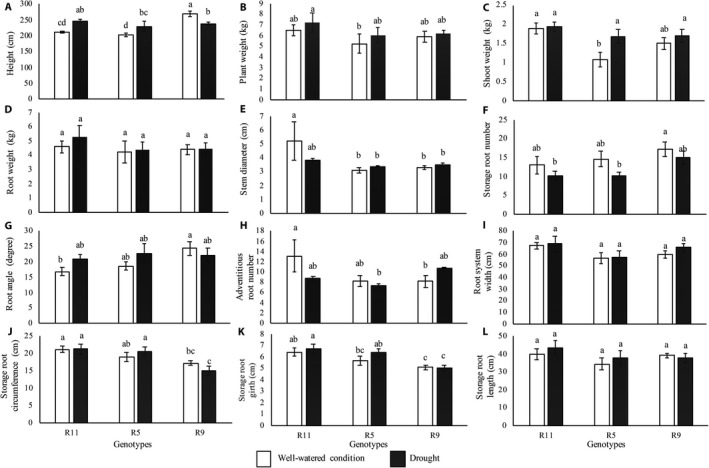
Shoot and root traits of three cassava varieties grown in the field under well‐watered and drought conditions at 12 months after planting. (A) Height, (B) plant weight, (C) shoot weight, (D) root weight, (E) stem diameter, (F) tuber number, (G) root angle, (H) adventitious root number, (I) root system width, (J) circumference, (K) storage root girth, (L) storage root length.

**Figure 7:**
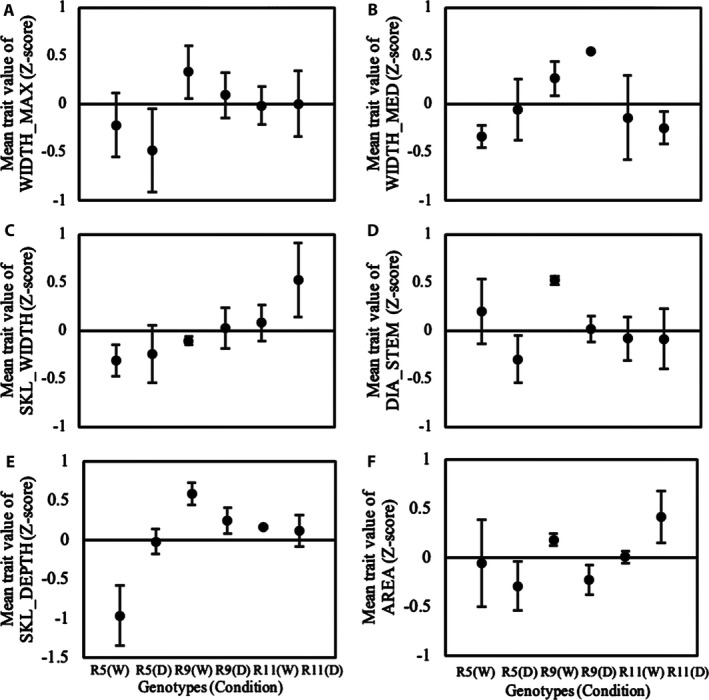
DIRT trait comparison for R5, R9, and R11. The plants were harvested at 12 months after planting in the field under well‐watered conditions (W) and drought conditions (D). (A) WIDTH_MAX, (B) WIDTH_MED, (C) SKL_WIDTH, (D) DIA_STEM, (E) SKL_DEPTH, and (F) AREA traits. Data were made comparable through normalization of mean trait values (*Z*‐score). Error bars represent the standard error of the mean for each treatment and genotype category that corresponds to a particular trait.

In the field, drought significantly reduced average storage root number by 21%. Genotypes responded to drought differently. R9 significantly decreased height by 12%, whereas R11 and R5 increased height under drought by 14% and 11%, respectively *(*Fig. 6A*)*. R9 and R11 maintained shoot weight, whereas R5 increased shoot weight by 36% under drought *(*Fig. 6C*)*. Although differences among root traits such as root system width and depth could not be captured by manual measurements *(*Fig. 6I, L*)*, DIRT successfully distinguished the differences among genotypes *(*Fig. 7 *)*. In the well‐watered treatment, R5 had the lowest SKL_DEPTH compared to R11 and R9 *(*Fig. 7E*)*. However, SKL_DEPTH of R5 was significantly increased under drought, which suggests that the root system became deeper compared to well‐watered treatments. Among genotypes, R9 had the highest root system width *(*WIDTH_MAX*)* and root depth *(*SKL_DEPTH*)* in all conditions *(*Fig. 7A, E*)*, as well as the highest storage root and adventitious root number under drought (Figs. 6F, 7H).

## DISCUSSION

We developed cassava root phenotyping protocols combining manual measurements and automatic DIRT measurements. Several root characteristics were identified and characterized (Table 1). These traits potentially influence cassava development under drought, volume of soil exploration, photosynthate accumulation patterns, and root yield. Phenotyping agronomically relevant traits of cassava is challenging because of its complicated root system and large size. However, cassava root shovelomics may be less time‐consuming compared to other species because most cassava cultivation is in sandy soils (USDA, NRCS, 2019) where the root system can easily be cleaned off using a paintbrush.

We demonstrated that DIRT applied to cassava can distinguish all genotypes, including several manually accessible traits such as width and depth as well as inaccessible traits such as D and DS values. Such shape descriptors give insight into depth development of the root systems; these traits had not previously been investigated in cassava and will provide insight into developmental traits that impact plant performance. Furthermore, DIRT allows for relatively high‐throughput measurements of the whole root structure. Significant correlations were found between data obtained from manual measurements and DIRT, such as those of root system width and root weight. This is important because root system width and root weight are critical denominators of root yield. Hence, our results suggest that the platform can facilitate genetic analysis with genome‐wide association studies (GWAS) and quantitative trait loci mapping (QTL) and is feasible for large‐scale selection in breeding programs. Most importantly, our study can be replicated by researchers around the world because of the free availability of DIRT.

Cassava expresses substantial genotypic variation in root traits. On average, the ranges of traits observed in our study are consistent with those reported by others. Our germplasm had longer adventitious root length at three months after transplanting than 28 cassava varieties in a pot system reported by Subere et al. (2009). This indicates opportunities for cassava breeders to use Thai germplasm as donors for increased adventitious root length in cassava breeding programs.

Drought significantly suppressed cassava shoot and root growth. In the pot system, only three cassava genotypes (R5, HB60, and KU50) formed small storage roots under drought. These storage roots were long and thin and had high storage root length : girth ratio (Table 2). Among different genotypes, KU50 increased total root number by 25% and enhanced the number of adventitious basal roots by more than 50%. Moreover, KU50 had the highest shoot dry weight among all genotypes under drought. In contrast, R11, which had the greatest total root number in well‐watered conditions, had a substantially reduced number of adventitious roots (by a factor of 3) and had the lowest shoot dry weight among cassava genotypes under drought. Maintaining adventitious roots is related to enhancing growth and yield performance, as well as enabling cassava to revive quickly after rewatering (Subere et al., 2009). This is also evidenced by a positive correlation between adventitious root number and biomass, including shoot dry weight (*r* = 0.44, *P* < 0.05), root dry weight (*r* = 0.70, *P* < 0.001), and plant dry weight (*r* = 0.37, *P* < 0.05), in the pot system and by the high storage root number found in genotype R9 when grown in the field under drought conditions. In other crop species such as maize, however, an increase in adventitious root number means less carbon and energy are allocated to produce deeper rooting and sustain shoot growth and yield (Saengwilai et al., 2014b). Different adaptive strategies such as shoot growth arrest, early stomata closure, and shedding of old leaves have been shown to be utilized by cassava (Zhao et al., 2017). These strategies could possibly balance the metabolic costs of increased adventitious root production, resulting in an overall improvement of plant growth under drought.

Several lines of evidence suggest that increased root growth angle is key for drought adaptation. For example, variation in several root traits of rice, including root growth angle, were linked to rice crops grown in flooded fields and in fields without flooding treatment (Saengwilai et al., 2018). Similarly, the *DEEPER ROOTING 1* (*DRO1*) gene was shown to increase root growth angle in rice to compensate for drought by increasing rooting depth (Uga et al., 2013). In maize, increased root growth angle was shown to be an adaptive strategy for acquisition of deep soil resources, particularly nitrate and water in poor soil and drought conditions (Lynch, 2013). In cassava, the physiological utility of root growth angle has not yet been shown. Our results showed that cassava had a very shallow root system compared to other crop species. The benefit of steep root growth angle for drought was not conclusive because the range of variation of root angle in this study was very small. It is also important to note that capturing root growth angle in mature cassava plants is challenging. In our study, root angle is the only trait that showed no correlation when compared using manual measurement and DIRT analysis. The DIRT analysis revealed shortcomings in the execution of the imaging protocol and difficulties in accessing the angle manually because of the complex arrangement of storage roots. An angle variation of about 10–15 degrees was introduced through root placement, because the radial arrangement of the storage roots allows the root to fall over on the stem. As a result, the projection of the 3D root structure onto the image plane distorts the angle measured in the image. For the manual measurement, it was difficult to identify a representative rooting angle because there is significant variation of present angles per storage root. Consequently, hardly any correlation could be found between manual and DIRT measurement of root angle in this study. Further improvements, such as using a clamp to hold the root system and repositioning the camera to capture images from a side angle, may help to alleviate these problems.

In order to further improve highly drought‐tolerant cassava genotypes, it is essential to develop a cassava root phenotyping protocol and platform and to provide more information about phenotypic variation of root systems among Thai cassava genotypes. In our experiment, adventitious root traits are suggested as a new breeding target to enhance water‐use efficiency. Moreover, molecular plant breeders could benefit from the phenotyping platform to obtain trait measurements applicable for GWAS and QTL analysis to facilitate marker‐assisted selection (Vogel, 2014). In moving forward, data sharing and recombination are important, because collecting field data sets that use the potential of high‐throughput and high‐resolution phenotyping with many repetitions per genotype is laborious and expensive due to the initial manual excavation process. Free accessibility of collected data sets is key to tap the potential of large data sets that provide a plethora of information that can only be revealed in community efforts. As a result, breeding projects for water and nutrient efficiency that do not require expensive field research facilities are enabled and can be targeted to the needs of smallholder farmers who face the constraints of low soil fertility and drought. Therefore, our phenotyping protocol can be useful for collecting data for low‐cost breeding and genetic research.

## Data Availability

The images and data that support the findings of this study are openly available on CyVerse Data Commons (as Saengwilai_Cassava_2019; https://doi.org/10.25739/ej8x-3b24).
